# A Novel Role for D-Alanylation of Lipoteichoic Acid of *Enterococcus faecalis* in Urinary Tract Infection

**DOI:** 10.1371/journal.pone.0107827

**Published:** 2014-10-08

**Authors:** Dominique Wobser, Liaqat Ali, Elisabeth Grohmann, Johannes Huebner, Türkan Sakinc

**Affiliations:** 1 Division of Infectious Diseases, Department of Internal Medicine II, University Hospital Freiburg, Freiburg, Germany; 2 Faculty of Biology, Albert Ludwigs University of Freiburg, Freiburg, Germany; 3 Division of Pediatric Infectious Diseases, Dr. Von Hauner Children's Hospital, Ludwig Maximilian University Munich, Munich, Germany; University of Rochester Medical Center, United States of America

## Abstract

**Background:**

Enterococci are the third most common cause of healthcare-associated infections, which include urinary tract infections, bacteremia and endocarditis. Cell-surface structures such as lipoteichoic acid (LTA) have been poorly examined in *E. faecalis*, especially with respect to urinary tract infections (UTIs). The *dlt* operon is responsible for the D-alanylation of LTA and includes the gene *dlt*A, which encodes the D-alanyl carrier protein ligase (Dcl). The involvement of LTA in UTI infection by *E. faecalis* has not been studied so far. Here, we examined the role of teichoic acid alanylation in the adhesion of enterococci to uroepithelial cells.

**Results:**

In a mouse model of urinary tract infection, we showed that *E. faecalis* 12030Δ*dlt*A mutant colonizes uroepithelial surfaces more efficiently than wild type bacteria. We also demonstrated that this mutant adhered four fold better to human bladder carcinoma cell line T24 compared to the wild type strain. Bacterial adherence could be significantly inhibited by purified lipoteichoic acid (LTA) and inhibition was specific.

**Conclusion:**

In contrast to bacteraemia model and adherence to colon surfaces, *E. faecalis* 12030Δ*dlt*A mutant colonized uroepithelial surfaces more efficiently than wild-type bacteria. In the case of the uroepithelial surface the adherence to specific host cells could be prevented by purified LTA. Our results therefore suggest a novel function of alanylation of LTA in *E. faecalis*.

## Introduction

Enterococci are the third most common cause of healthcare-associated infections, which include urinary tract infections, bacteremia and endocarditis. Risk groups for invasive enterococcal infections include neonates, Intensive Care Unit (ICU) patients and immunocompromised hosts [1]. The increasing identification of enterococcal strains resistant to multiple antibiotics in recent decades represents a serious threat to therapy and emphasizes the need for a better understanding of the pathogenicity of these microbes.

Bacterial adherence is an important step in the disease process by facilitating colonization and translocation across the mucosal barrier, which eventually results in systemic dissemination within the host [2]. Like other Gram-positive bacteria (e.g., *Staphylococcus aureus*), the surface of *E. faecalis* is rich in exposed adhesins that mediate binding to human receptors or to various components of the extracellular matrix (ECM); they are thus considered a member of MSCRAMM-microbial surface component–recognizing adhesive matrix molecules [3]. Basically, MSCRAMMs are cell wall–anchored surface proteins that have characteristic immunoglobulin-like folds [4].

Attachment of microorganisms to mucosal surfaces of the urinary tract is important for the pathogenesis of UTI, because the mechanical removal of colonizing bacteria by the urine flow is an important innate defense mechanism. In the process of bacterial cell adherence, infectious agents interfere with specific molecules on epithelial cells [5]–[6]. Furthermore, it has been shown that the tendency of certain bacteria to infect specific tissues is often related to their ability to adhere to a specific target cell.

While UTI-specific virulence factors of *E. coli* have been studied extensively, relatively little is known about *E. faecalis* cell-surface structures with respect to UTIs [7–10 & 11]. In a model of ascending UTI, the presence of an enterococcal surface protein encoded by an acquired gene was shown to increase the persistence of bacteria in the urinary bladders of mice [7] whereas [9] demonstrated that *E. faecalis* has greater tropism for the kidneys. Using a similar model, the MSCRAMM adhesion of collagen of *E. faecalis* (Ace) was recently identified as a putative virulence factor involved in colonization of renal tissue [12]–[13]. In another study, Torelli *et al.* reported that a PavA-like fibronectin-binding protein in *E. faecalis* called enterococcal fibronectin-binding protein A (EfbA) contributes to the pathogenesis of enterococcal UTIs. The EfbA mutant shows attenuated colonization in a mouse model of mounting UTI [11]. Ebp, a biofilm-associated pilus, produces immunogenic and pleomorphic pili [14] and also plays a role in UTI pathogenesis [15].

Generally, the functions of most *E. faecalis* cell surface structures have been poorly investigated, especially with respect to UTIs [7]–[10], [16]. The enterococcal cell wall contains peptidoglycan (PG) and a number of accessory cell-wall polysaccharides and glycoconjugates such as LTA wall teichoic acid (WTA), a rhamnose-containing cell-wall polysaccharide *epa* (enterococcal polysaccharide antigen) [17]–[20] and a diheteroglycan [21]. Lipoteichoic acid (LTA) is an amphiphilic polymer consisting of polyglycerolphosphate and a glycolipid anchor inserted into the cell membrane [22]–[23]. The *dlt* operon includes four genes and is responsible for the D-alanylation of LTA [24]. One of these genes, *dlt*A, encodes the D-alanyl carrier protein ligase (Dcl), which activates the D-alanine and ligates it to the D-alanyl carrier protein (Dcp). The involvement of LTA in UTI infection by *E. faecalis* has not been studied. Here we examined the role of teichoic acid alanylation in the adhesion of enterococci to uroepithelial cells.

## Materials and Methods

### Cell culture

T24 human bladder carcinoma cells (Cell Line Service, Eppelheim, Germany) were cultured in Dulbeccós Modified Eaglés Medium (DMEM, Sigma Aldrich), nutrient mixture F-12 Ham, supplemented with 5% fetal bovine serum (FBS Superior, Biochrom AG) in a humidified 5% CO_2_ atmosphere at 37°C.

### Bacterial strains and chemicals

*E. faecalis* 12030 strain is a clinical isolate obtained in Cleveland, OH. It is a strong biofilm producer and is opsonized by antibodies against its LTA [25]. *E. faecalis* 12030Δ*dlt*A mutant and its complemented strain *E. faecalis* 12030Δ*dlt*Acompl were kindly provided by C. Theilacker and F. Fabretti. All bacterial strains were grown at 37°C without agitation in Caso Bouillon (Carl Roth), and reagents were obtained from Sigma.

### LTA extraction

LTA from *E. faecalis* 12030 wild type were isolated as described [26] and with modifications by Theilacker *et al.*, 2006 [25]. Bacterial cells were grown in tryptic soy broth, harvested after 3 h and resuspended in 0.1 M citrate buffer (pH 4.7). The harvested cells were disrupted using glass beads (Beadbeater; Glenn Mills, Clifton, NJ) and stirred with an equal volume of n-butanol for 30 min at room temperature. After centrifugation, the aqueous layer was dialyzed against 0.1 M ammonium acetate (pH 4.7) and lyophilized. The material was redissolved in 15% n-propanol in 0.1 M ammonium acetate (pH 4.7) and applied to an octyl Sepharose column for hydrophobic interaction chromatography. Bound material was eluted with a gradient of 15–80% n-propanol. Fractions were assayed for total phosphorus and by immunoblot assay [26].

### Invasion/Adherence assay

For the adherence assay, cells were cultivated in 24-well plates to a density of approximately 1×10^5^ cells/well for 16 h. Bacteria were inoculated at (OD_600 nm_ ∼ 0.1) and grown to mid-log phase (OD_600 nm_ ∼ 0.4) at 37°C in Caso Bouillon. T24 cells were incubated with bacteria for 2 h at a multiplicity of infection of 100∶1. After infection of the monolayer, human bladder carcinoma cells were washed five times with bovine serum albumin (PBS, Biochrom AG) and lysed with DMEM F-12 Ham + 5% FBS + 0.25% Triton-X100 buffer for 15 min. Surface adherent and intracellular bacteria were enumerated by quantitative bacterial counts. Six replicates (wells) of each stimulation were prepared and the full experiment was repeated 3 times.

### Inhibition assays

For inhibition of enterococcal binding to T24 cells, purified LTA (10–500 µg/mL) from wild type *E. faecalis* 12030 was added to the cells 30 min before infection. The assay was then performed as described above.

### Mouse Urinary Tract Infection model

Female BALB/C mice (6–8 weeks old, Charles River, Sulzfeld, Germany) were used for the experiments. To prepare the inoculum, bacteria were grown in 5 mL for ∼ 16 h at 37°C with gentle shaking. For inoculum preparation the overnight culture was diluted 1∶100 in 50 mL fresh medium and grown to an OD_600nm_ ∼ 0.3 at 37°C with shaking. The cells were pelleted for 10 min (6.000×g at 10°C) and resuspended in half of the volume of 0.9% saline. Further dilutions were prepared in Caso Bouillon and then plated to determine the actual inoculum. Before starting the experiment, the bladder of the mouse was emptied by catheter (20 mm). Subsequently, isoflurane-anesthetized mice were infected via urethral catheterization with 100 µL of the bacterial suspension (3–5×10^8^ CFU/mL). After 2 h the same amount of bacteria was injected again to achieve consistent infection. The urethral catheter was removed immediately after this step, and all animals had free access to food and water during the course of study. Mice were euthanized by CO_2_ inhalation at 24 h and 48 h after transurethral challenge. The urinary bladder and kidney pairs were excised, weighed, and homogenized in 1 mL of 0.9% saline + 0.025% Triton X-100, and dilutions were plated onto Caso Agar without antibiotics. The CFU/100 mg of bacteria for each tissue (bladder or kidney) was calculated for each animal.

### Statistical analysis

If not stated otherwise, the experiments were repeated three times. All cell culture tests were made by multigroup comparisons using ANOVA (PRISM4, GraphPad software). P value less than 0.05 was considered significant. Comparisons among groups were made using the Mann-Whitney U test. P value less than 0.05 was considered significant.

### Ethics Statement

All animal experiments were performed in compliance with the German animal protection law (TierSchG). The mice were housed and handled in accordance with good animal practice as defined by FELASA and the national animal welfare body GV-SOLAS. The animal welfare committees of the University of Freiburg (Regierungspräsidium Freiburg Az 35/9185.81/G-11/118) approved all animal experiments.

## Results

### *E. faecalis* 12030Δ*dlt*A mutant shows strong adherence to uroepithelial cells

**Figure 1:**
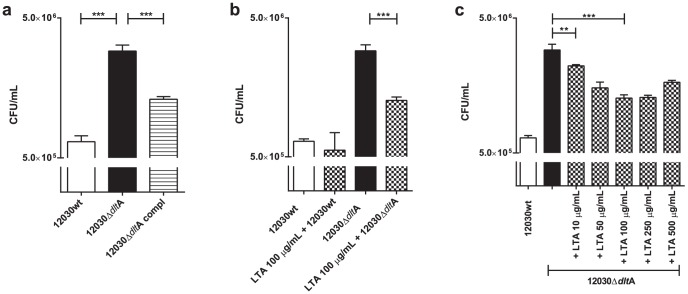
Attachment to T24 cells. (**A**) *E. faecalis* 12030 wild type, *E. faecalis* 12030Δ*dlt*A mutant and the *E. faecalis* 12030Δ*dlt*A complemented strain were tested for their ability to adhere to and/or invade T24 uroepithelial cells. Bacterial titers in logarithmic scale (log scale). T24 cells were cultivated in 24-well plates to a density of 1×10^5^ cells/well and incubated for 2 h with bacteria grown to mid-log phase (OD_600nm_ ∼ 0.4) at a bacteria-to-cell ratio of 100:1. Total cell-associated bacteria include surface-adherent and intracellular bacteria. Bars represent average ± S.E. The *E. faecalis* 12030Δ*dlt*A mutant shows strongly increased adherence to uroepithelial cells compared to the wild type (p<0.001). **B**) Treatment of cells with purified LTA. *E. faecalis* 12030 wild type and the *E. faecalis* 12030Δ*dlt*A mutant were tested. Concentration of purified LTA was 100 µg/mL. The adherence to uroepithelial cells was significantly reduced by *E. faecalis* 12030Δ*dlt*A mutant (p<0.01) after LTA treatment and no differences were obtained by wild type strain. In each experiment 6 replicates (wells) of each stimulation were prepared. The full experiment was repeated 3 times. Multigroup comparisons were made by ANOVA (PRISM4, GraphPad software). P-values of <0.05 (*), <0.005 (**) and <0.0005 (***) were considered statistically significant. **C**) Dose-dependent reduction in adhesion using purified LTA. *E. faecalis* 12030Δ*dlt*A mutant were tested with increasing concentrations of LTA (10 – 500 µg/mL).

To investigate the importance of LTA D-alanylation to in vitro adherence to uroepithelia, we used T24 human bladder cell lines, *E. faecalis* 12030Δ*dlt*A mutant and the wild type *E. faecalis* 12030. As control, the *E. faecalis* 12030Δ*dlt*A complemented strain was used. As shown in Figure 1a, compared to the wild type strain (0.6×10^6^ CFU/mL), the *E. faecalis* 12030Δ*dlt*A mutant showed up to four fold increased adherence (2.9×10^6^ CFU/mL, p<0.001) to uroepithelial cells. The complemented mutant strain *E. faecalis* 12030Δ*dlt*Acompl showed a partially restored effect of the mutation (1.2×10^6^ CFU/mL, p<0.001).

### Bacterial adherence could be inhibited by purified LTA

To study the specific interaction between bacteria and a still-unknown receptor, T24 cells were first incubated with 100 µg/mL purified LTA from wild type *E. faecalis* 12030 strain to saturate binding sites and subsequently they were inoculated with bacteria. As shown in Figure 1b, the *E. faecalis* 12030Δ*dltA* mutant with LTA treatment showed significantly less adherence (1.25×10^6^ CFU/mL, p<0.01) to the bladder epithelial cell line as compared to without LTA treatment (2.9×10^6^ CFU/mL). In the wild type, no significant differences in adherence properties were observed using purified LTA and a dose dependent reduction of adherence using LTA could also demonstrated in Figure 1c.

### *E. faecalis* 12030Δ*dlt*A colonizes uroepithelial surfaces more efficiently than wild type bacteria

**Figure 2:**
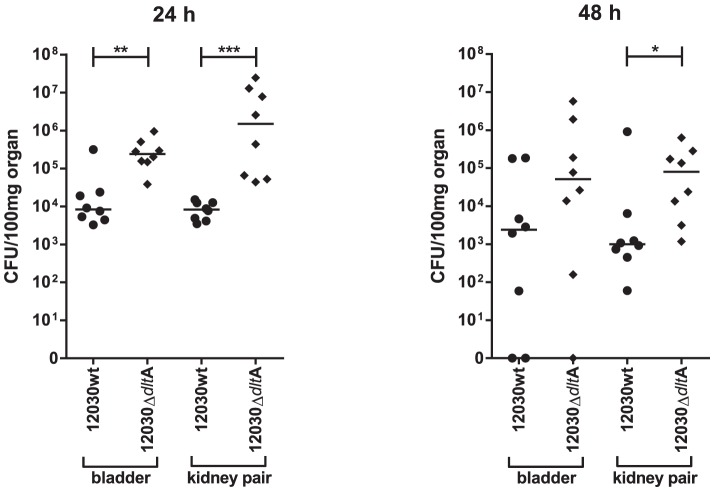
Monoinfection with wild-type *E. faecalis* 12030 (12030 wt) and mutant *E. faecalis* 12030Δ*dlt*A. Bacterial titers in logarithmic scale (log scale). Data are from 8 mice infected with 3–5×10^8^ CFU; results are expressed as CFU per 100 mg of kidney and bladder homogenates 24 h and 48 h after transurethral challenge. The CFU from both kidneys were averaged. A value of 1 CFU was assigned to kidneys without bacteria. Circles represent wild-type *E. faecalis* 12030, squares represent *E. faecalis* 12030Δ*dlt*A. Horizontal bars represent the geometric mean. Values that are significantly different by the Mann-Whitney U test are indicated as follows: P-value of <0.05 (*), <0.005 (**) and <0.0005 (***).

To study the effect of lipoteichoic acid on urinary tract infection, a modified mouse urinary infection model was used [9]. The wild type strain *E. faecalis* 12030 and the mutant *E. faecalis* 12030Δ*dlt*A were tested. The bacterial counts in the bladder and in both kidneys were determined after 24 h and 48 h of incubation as described elsewhere [9]. As shown in Figure 2 after 24 h, the mutant colonized the bladder (4×10^5^ CFU/100 mg) significantly better compared to the wild type strain (9×10^3^ CFU/100 mg), i.e. up to 1.5 log more bacteria (p<0.001). This difference in colonization behaviour between mutant and wild type strains was 2.4 logs higher in kidneys (p<0.001). The mutant showed 3×10^6^ CFU/100 mg tissue compared to wild type 9×10^3^ CFU/100 mg. We observed colonization differences between mutant and wild type strains to bladder and kidney epithelia after the prolonged incubation time of 48 h. A statistically significant difference (p<0.05) was observed only in kidneys. Here the mutant colonized up to 9×10^5^ CFU/100 mg tissue whereas the wild type achieved only 1×10^3^ CFU/100 mg. In fact, the *E. faecalis* 12030Δ*dlt*A mutant colonized the kidneys at both time points considerably better than the wild-type strain.

## Discussion

Attachment of microorganisms to mucosal surfaces of the urinary tract is important for the pathogenesis of UTI because the mechanical removal of colonizing bacteria by the urine flow is an important innate defense mechanism. In the process of bacterial cell adherence, infectious agents interfere with specific molecules on epithelial cells [17], [20], [5]–[6], [27], [28]. While UTI-specific virulence factors of *E. coli* have been studied extensively, relatively little is known about *E. faecalis* cell-surface structures with respect to UTIs [7–10 & 11].

In a model of ascending UTI, the presence of an enterococcal surface protein was shown to increase the persistence of bacteria in the urinary bladders of mice without histological changes [7] whereas Kau *et al.* demonstrated that *E. faecalis* has greater tropism for the kidneys [9]. Using a similar model, the MSCRAMMs of *E. faecalis* such as collagen adhesion protein (Ace) [12]–[13], PavA-like fibronectin-binding protein (EfbA) [11], and a biofilm-associated pilus (Ebp) [14], [15] contributed to the pathogenesis of enterococcal UTIs.

The D-Ala-LTA formation racks up several functions, such as cationic homeostasis maintenance, integration of metal cations and inflection of autolytic actions and several other electromechanical properties of the bacterial envelope when the surface of gram-positive bacteria is exposed to disparate microenvironments. The cell wall of gram-positive bacteria contains different types of anionic molecules. Two different teichoic acids are present on the surface of gram-positive bacteria: lipoteichoic acid (LTA), an amphiphilic polymer non-covalently inserted into the cellular membrane, and wall teichoic acid (WTA), covalently linked to the peptidoglycan of the cell wall. These two molecules are synthesized by separate biosynthetic pathways that have been well characterized in *Bacillus subtilis* and other gram-positive bacteria [29]. Nevertheless, D-alanyl-ester substituents of WTA originally derive from LTA and are later transferred to WTA by transacylation. It is a single *dlt* operon encoding the genes responsible for the D-alanine incorporation [30]–[32].

The *dltA* operon has been studied in several gram-positive bacteria revealing an identical organization [33]. Bacteria with mutations in the *dlt* operon showed a variety of phenotypic changes that could be attributed to the resulting charge modification of their cell surface. The lack of D-alanine esters resulted in a stronger negative net charge, because D-alanine esters introduce positively charged groups into the otherwise negatively charged teichoic acids [34]. In *B. subtilis* and *S. aureus,* the absence of D-alanine has been shown to cause alterations in the activity of autolytic enzymes [35], [36]. Also possible is an altered host immune response, leading to enhanced proliferation/persistence.

In this work, the *E. faecalis* 12030Δ*dlt*A mutant has been investigated with regard to adherence to T24 bladder carcinoma cells. Surprisingly, we observed that it displayed more than four-fold increased adherence to human bladder carcinoma cells compared to the wild-type strain. We could partially restore this adherence to 1.310^6^ CFU/mL using a complemented mutant strain (Figure 1a). Moreover, using purified LTA it was possible to significantly inhibit adherence to human bladder cells, demonstrating the specificity of binding (Figure 1b). A dose dependent reduction of adherence using LTA could also be demonstrated (data not shown). Interestingly, in contrast to our previous data, which showed that the same mutant exhibits less binding to Caco2 colonic epithelial cells than wild-type bacteria, here we obtained higher adherence to uroepithelial cells [3], [25], [37]. In addition, these data are in contrast to previous studies using *S. aureus* and *Listeria monocytogenes*, in which elimination of D-alanylation of LTA impaired adherence to mammalian cells [38]–[40].

To assess the relevance of these results and the differences in vivo, a modified mouse urinary infection model was used; our results confirmed that the *E. faecalis* 12030Δ*dlt*A mutant colonized the kidneys significantly better than the wild-type strain after 24 and 48 h (Figure 2). In addition, the mutant colonized the bladder significantly better after 24 and 48 h albeit the differences observed at 48 h were not statistically significant. This is assumed by the fact that *E. faecalis* usually does not persist in the bladder, resulting in decrease number of colony counts, because of clearance of bacteria from the bladder by mechanical forces of urine flow.

However, the same *E. faecalis* 12030Δ*dlt*A mutant demonstrated less colonization in a mouse sepsis model [37]. This disparate finding suggests the existence of specific receptors on bladder and/or uroepithelial cell surfaces. Our results therefore show that D-alanylation of LTA can decrease adherence to specific host cells and therefore suggest a novel function of alanylation of LTA in *E. faecalis*. Differential expression of the *dlt* locus may allow enterococci to adapt to specific ecological niches.
